# Identification of Autophagy as a Functional Target Suitable for the Pharmacological Treatment of Mitochondrial Membrane Protein-Associated Neurodegeneration (MPAN) In Vitro

**DOI:** 10.3390/pharmaceutics15010267

**Published:** 2023-01-12

**Authors:** Enrica Zanuttigh, Kevork Derderian, Miriam A. Güra, Arie Geerlof, Ivano Di Meo, Chiara Cavestro, Stefan Hempfling, Stephanie Ortiz-Collazos, Mario Mauthe, Tomasz Kmieć, Eugenia Cammarota, Maria Carla Panzeri, Thomas Klopstock, Michael Sattler, Juliane Winkelmann, Ana C. Messias, Arcangela Iuso

**Affiliations:** 1 Institute of Neurogenomics, Helmholtz Zentrum München, 85764 Neuherberg, Germany; 2 Protein Expression and Purification Facility, Institute of Structural Biology, Molecular Targets and Therapeutics Center, Helmholtz Zentrum München, 85764 Neuherberg, Germany; 3 Medical Genetics and Neurogenetics Unit, Fondazione IRCCS Istituto Neurologico Carlo Besta, 20126 Milan, Italy; 4 Institute of Structural Biology, Molecular Targets and Therapeutics Center, Helmholtz Zentrum München, 85764 Neuherberg, Germany; 5 Bavarian NMR Centre, Department of Bioscience, School of Natural Sciences, Technical University of Munich, 85747 Garching, Germany; 6 Molecular Cell Biology Section, Department of Biomedical Sciences of Cells & Systems, University of Groningen, University Medical Center Groningen, 9713 AV Groningen, The Netherlands; 7 Expertise Center Movement Disorders Groningen, University Medical Center Groningen, 9713 AV Groningen, The Netherlands; 8 Department of Neurology and Epileptology, The Children’s Memorial Health Institute, 04-730 Warsaw, Poland; 9 Alembic, Experimental Imaging Center, IRCCS San Raffaele Hospital, 20132 Milan, Italy; 10 Department of Neurology, Friedrich-Baur-Institute, University Hospital of the Ludwig-Maximilians-University (LMU), 80336 Munich, Germany; 11 Munich Cluster for Systems Neurology (SyNergy), 81377 Munich, Germany; 12 German Center for Neurodegenerative Diseases (DZNE), 81377 Munich, Germany; 13 Institute of Human Genetics, Klinikum Rechts der Isar, Technical University of Munich, 81675 Munich, Germany

**Keywords:** C19orf12, mitochondria membrane protein-associated neurodegeneration (MPAN), neurodegeneration with brain iron accumulation (NBIA), autophagy, LC3, carbamazepine, ABT-737, LY294002, oridonin, paroxetine

## Abstract

Mitochondrial membrane protein-associated neurodegeneration (MPAN) is a relentlessly progressive neurodegenerative disorder caused by mutations in the *C19orf12* gene. C19orf12 has been implicated in playing a role in lipid metabolism, mitochondrial function, and autophagy, however, the precise functions remain unknown. To identify new robust cellular targets for small compound treatments, we evaluated reported mitochondrial function alterations, cellular signaling, and autophagy in a large cohort of MPAN patients and control fibroblasts. We found no consistent alteration of mitochondrial functions or cellular signaling messengers in MPAN fibroblasts. In contrast, we found that autophagy initiation is consistently impaired in MPAN fibroblasts and show that *C19orf12* expression correlates with the amount of LC3 puncta, an autophagy marker. Finally, we screened 14 different autophagy modulators to test which can restore this autophagy defect. Amongst these compounds, carbamazepine, ABT-737, LY294002, oridonin, and paroxetine could restore LC3 puncta in the MPAN fibroblasts, identifying them as novel potential therapeutic compounds to treat MPAN. In summary, our study confirms a role for C19orf12 in autophagy, proposes LC3 puncta as a functionally robust and consistent readout for testing compounds, and pinpoints potential therapeutic compounds for MPAN.

## 1. Introduction

Mitochondrial membrane protein-associated neurodegeneration (MPAN) is a rare form of neurodegeneration with brain iron accumulation (NBIA) [1]. Patients with MPAN present with progressive spastic paraparesis, psychiatric disturbances, and slow cognitive decline, variably accompanied by motor neuropathy, extrapyramidal, and visual symptoms during the disease course [2]. MPAN is associated with autosomal recessive (AR) and dominant (AD) pathogenic variants in the gene *C19orf12* (*614297) [2,3,4,5,6]. The encoded protein is evolutionarily conserved [2]. Downregulation of the two *C19orf12* orthologs in fruit flies leads to neurodegeneration comparable to humans [7]. Additionally, in zebrafish embryos, the downregulation of *C19orf12* negatively affects neuronal and muscular development [8].

In humans, the gene encodes a membrane-associated protein that localizes to mitochondria, endoplasmic reticulum (ER), and endoplasmic reticulum–mitochondria contact sites [2,9,10]. According to the public database GTEx (Analysis Release V8) [11], *C19orf12* is ubiquitously expressed, with highest expression levels in adipose tissue, brain, and muscle.

So far, the function of C19orf12 is unknown, though the protein has been associated with a potential role in lipid metabolism. In 2011, Hartig et al. showed that *C19orf12* expression in whole blood is co-regulated with genes involved in fatty acid biogenesis and valine, leucine, and isoleucine degradation. The authors also showed that *C19orf12* expression increases continuously during the differentiation of healthy pre-adipocytes into fully mature adipocytes [2]. A recent study in fruit flies investigated the functional interplay between C19orf12 and PLA2G6. Over-expression of *C19orf12* in *PLA2G6*-deficient flies rescued acyl-chain shortening in phospholipids caused by loss of *PLA2G6* [12]. Furthermore, Drecourt et al. revealed that mutations in *C19orf12* and other NBIA genes are associated with reduced palmitoylation of the transferrin receptor, leading to its impaired recycling and elevated cytosolic iron accumulation in patients’ fibroblasts [13].

A role for C19orf12 in autophagy has been proposed by Venco et al. [10]. The study reported that over-expression of *C19orf12* stimulates lipidation of the autophagy marker LC3-I to its active form LC3-II. The authors also showed that fibroblasts derived from one MPAN patient carrying the homozygous G58S variant were more susceptible to oxidative stress-induced cell death and exhibited increased mitochondrial calcium compared with control cells, suggesting that the mutation impairs mitochondrial function and calcium homeostasis [10].

More recently, both recessive and dominant pathogenic variants in *C19orf12* have been associated with impaired mitochondrial respiration, reduced mitochondrial length and cellular adenosine 5′-triphosphate (ATP), as well as increased iron, reactive oxygen species (ROS), and lipid peroxidation, conferring MPAN cells an increased susceptibility to ferroptosis, which was rescued upon treatment with the iron chelator DFO [14].

Considering the variety of phenotypes associated with C19orf12 deficiency and the need to identify a robust functional target for the assessment of therapeutic options for MPAN patients and provide them with rational treatment, we re-evaluated most of the reported phenotypes in a larger sample cohort.

We provide evidence that impaired autophagy is the only significant readout in MPAN fibroblasts and can be used to screen small molecules with potential therapeutic effects in MPAN.

## 2. Materials and Methods

### 2.1. Fibroblasts Cell Lines and Culturing Condition

A list of primary skin fibroblasts used in this study and their variants in *C19orf12* can be found in Table S1. Control cells over-expressing C19orf12 (33281-T-C19orf12) were generated as described in [15] by cloning the C19orf12 isoform NM_001031726.3 in the pLenti6.3/V5™-TOPO plasmid (Invitrogen, Waltham, MA, USA, K531520).

Commercial cells included NHDF (Lonza, Basel, Switzerland, CC-2509), HEK293FT (Invitrogen, R70007), HeLa, Hep G2, and SH-SY5Y (ATCC, Manassas, VA, USA).

Cells were cultured in high glucose Dulbecco’s Modified Eagle Medium (Life Technologies, Carlsbad, CA, USA, 41966029) supplemented with 10% fetal bovine serum (Life Technologies, 10270106), 50 U/mL penicillin/streptomycin (Life Technologies, 15070063), and 200 μM uridine (Sigma, St. Louis, MI, USA, U3750), at 37 °C in a 5% CO_2_ humidified atmosphere. All cell lines tested negative for mycoplasma contamination using the MycoAlert Mycoplasma detection kit (Lonza, LT07-118), according to the manufacturer’s instructions.

### 2.2. Protein Overexpression and Purification

#### 2.2.1. Preparation of Expression Constructs

Human C19orf12 long isoform (Q9NSK7-1; residues 1-152; 152 amino acids long), medium isoform (Q9NSK7-4; residues 12-152; 141 amino acids long), and short isoform (Q9NSK7-2; residues 79-152; 77 amino acids long) were amplified by PCR using Pfu polymerase and cloned into pETM-11, an expression vector containing an N-terminal His_6_-tag followed by a TEV protease cleavage site [16]. All expression constructs were verified by sequencing.

#### 2.2.2. Protein Expression and Purification

The constructs for the long and medium isoforms were transformed into *E. coli* strain Rosetta2 (DE3) and cultured at 20 °C in 2-L flasks containing 500 mL ZYM 5052 auto-induction medium [17], 100 µg/mL kanamycin, and 33 µg/mL chloramphenicol. The construct for the short isoform was transformed into *E. coli* strain Rosetta2 (DE3) pLysS and cultured in 2-L flasks containing 500 mL of ZYM 5052 and 100 µg/mL kanamycin, and 33 µg/mL chloramphenicol. Cells were harvested by centrifugation after reaching saturation.

For the purification of long and medium isoforms, cells from 1 L of culture were resuspended in 60 mL lysis buffer (50 mM Tris-HCl, 300 mM NaCl, 20 mM imidazole, 10 mM MgCl_2_, 10 µg/mL DNaseI, 1 mM AEBSF.HCl, 1 mg/mL lysozyme, 0.01% (*v*/*v*) 1-thioglycerol, pH 8.0) containing 1% (*w*/*v*) DDM, incubated for 10 min on the bench and lysed by sonication. The lysate was clarified by centrifugation (40,000× *g*) and filtration (0.45 µM). The supernatant was applied to a 3-mL Ni-NTA column (Qiagen, Hilden, Germany), equilibrated in binding buffer (50 mM Tris-HCl, 300 mM NaCl, 20 mM imidazole, 0.01% (*v*/*v*) 1-thioglycerol, 0.01% (*w*/*v*) DDM, pH 8.0). The column was washed 2 times with 25 mL binding buffer. Bound proteins were eluted with elution buffer (50 mM Tris-HCl, 300 mM NaCl, 300 mM imidazole, 0.01% (*w*/*v*) DDM, 0.01% (*v*/*v*) 1-thioglycerol, pH 8.0). Fractions containing the target proteins were pooled and dialyzed overnight at 4 °C against 1 L SEC buffer (50 mM Tris-HCl, 300 mM NaCl, 0.01% (*w*/*v*) DDM, 0.01% (*v*/*v*) 1-thioglycerol, pH 8.0) in the presence of His-tagged TEV protease in a 1:25 molar ratio (TEV:protein). As described above, the cleaved-off proteins were further purified by affinity chromatography, and the flow-through and protein-containing wash fractions were pooled and concentrated to less than 5 mL. This was subsequently subjected to size exclusion chromatography using a HiLoad Superdex 200 16/600 GL column (Cytiva, Emeryville, CA, USA), equilibrated in SEC buffer. The elution fractions containing the C19orf12 protein were collected and concentrated to approx. 10 mg/mL and stored at −80 °C.

For the purification of the short isoform, cells from 1 L of the culture were resuspended in 60 mL lysis buffer (50 mM Tris-HCl, 300 mM NaCl, 20 mM imidazole, 10 mM MgCl_2_, 10 µg/mL DNaseI, 1 mM AEBSF.HCl, 0.2% (*v*/*v*) NP-40, 1 mg/mL lysozyme, 0.01% (*v*/*v*) 1-thioglycerol, pH 8.0), and lysed by sonication. The lysate was clarified by centrifugation (40,000× *g*) and filtration (0.2 µM). The purification was performed as described above using buffers without DDM.

Protein concentrations were determined by measuring the absorbance at 280 nm using the extinction coefficient of 13980 (long and medium isoforms) and 8696.07 (short isoform) M^−1^·cm^−1^, respectively.

### 2.3. Multi-Well Plate-Based Analyses

#### 2.3.1. Quantification of Intracellular Calcium in Fibroblasts

Cells were seeded in black 96-well plates with black bottoms at a density of 20,000 cells/well and left to adhere overnight. The following day, intracellular calcium was measured using the Cal-520 AM kit (Abcam, Cambridge, UK, ab171868). Cells were washed with HHBS (AAT Bioquest, Pleasanton, CA, USA, 20011) and incubated with 10 µM Cal-520 in HHBS supplemented with 10% FBS for 90 min at 37 °C, followed by 30 min at room temperature. Afterward, half of the wells were treated for 3 min with 100 µM ATP (Sigma, A6419) in HHBS, while the other half were incubated with HHBS only. Fluorescence was measured using the microplate reader with the following parameters: excitation/emission = 490/525 nm.

Afterward, cells were washed with PBS (Life Technologies, 14190094) and mechanically lysed using 1× mammalian lysis buffer (Abcam, ab179835) freshly supplemented with protease inhibitors (Merck, Rahway, NJ, USA, 535140) by pipetting up and down and scraping the bottom of the wells with the pipette tips. After 15 min of incubation, cells were centrifuged at 435× *g* for 5 min, and the supernatant was transferred to a clean plate for protein quantification.

#### 2.3.2. Quantification of Intracellular ROS in Fibroblasts

Cells were seeded in black 96-well plates with black bottoms at 20,000 cells/well density and left to adhere overnight. The following day, ROS were measured using the DCFDA/H2DCFDA—Cellular ROS Assay Kit (Abcam, ab113851). Cells were washed once with 1× buffer, then incubated with 25 µM DCFDA in 1× buffer for 45 min at 37 °C. Afterward, cells were washed once with 1x buffer, and half of the wells were treated with 50 µM tBHP in 1× buffer supplemented with 10% FBS. Next, 1× buffer + 10% FBS was added to the other half of the wells. After 3 h of incubation at 37 °C, cells were washed with PBS, and fluorescence was measured in the microplate reader at excitation/emission = 485/535 nm. Cells were lysed for protein quantification.

#### 2.3.3. Quantification of Reduced Glutathione in Fibroblasts

Cells were seeded in transparent 96-well plates at a density of 20,000 cells/well and left to adhere overnight. The following day, cells were lysed, and protein concentration was measured with Bradford. Reduced glutathione in cell lysates was measured using GSH/GSSG Ratio Detection Assay Kit (Abcam, ab138881). A GSH standard curve was prepared according to the manufacturer’s instructions in a black 96-well plate, to which the cell lysates were added. The GSH Assay Mixture was added to each GSH standard and cell lysate sample. After 10 min of incubation, fluorescence was measured with the microplate reader at excitation/emission = 490/520 nm.

#### 2.3.4. Quantification of ATP in Fibroblasts

Cells were seeded in transparent 96-well plates at a density of 20,000 cells/well and left to adhere overnight. The following day, cells were lysed using boiling 100 mM Tris, 4 mM EDTA pH 7.5. After 2 min of incubation, cells were centrifuged at 1000× *g* for 1 min, and the supernatant was transferred to black 96-well plates. According to the manufacturer’s instructions, total ATP was quantified using ATP Bioluminescence Assay Kit CLS II (Roche, Basel, Switzerland, 11699695001). Luminescence was measured using the microplate reader at 10 s integration time.

#### 2.3.5. Oxygen Consumption Assay

Cells were seeded in Seahorse XF96 Cell culture microplates (Agilent Technologies, Santa Clara, CA, USA, 102416-100) at 20,000 cells/well density and left to adhere overnight. On the same day, Seahorse XF96 Sensor Cartridges (Agilent Technologies, 102416-100) were hydrated by adding sterile water to each well of the utility plate. The following day, water was removed, and Seahorse XF Calibrant solution (Agilent Technologies, 102416-100) was added to the Sensor Cartridge and incubated for 45–60 min in a 37 °C incubator without CO_2_. Cells were washed with medium (4.5 g/L glucose, 1 mM pyruvate, pH 7.4) and incubated with the same solution for 30 min in a 37 °C incubator without CO_2_. Oxygen consumption rate (OCR) was measured during 3 cycles of 2 min mixing, 2 min waiting, and 3 min measuring at the basal state and in the presence of 1 µM oligomycin, 0.4 µM FCCP, and 2 µM/2.5 µM of rotenone/antimycin A. At the end of the measurements, cells were washed with PBS and the plate was stored at −80 °C. The following day, the number of cells was counted using the CyQUANT™ Cell Proliferation Assay according to the manufacturer’s instructions.

Fluorescence values (i.e., calcium, ROS, and GSH) and luminescence values (i.e., ATP) were normalized to proteins with a Bradford assay (Bio-Rad, Hercules, CA, USA, 5000006) using a plate reader (Cytation 3, BioTek, Winooski, VT, USA). Oxygen consumption rates (OCR) were normalized to cell numbers with CyQUANT™ Cell Proliferation Assay (Invitrogen, C7026).

To control for inter-plate variability, control NHDF cells were seeded in each plate of each independent experiment. Within each plate, fluorescence, luminescence, and OCR values of control and MPAN samples were normalized to proteins or cell number normalized NHDF average values (i.e., fold change vs. NHDF).

In each assay, fold change values are shown in box plots. Within each box, horizontal black lines denote median values. Boxes extend from the 25th to the 75th percentile of each group’s distribution and the whiskers denote adjacent values. Dots outside of the whiskers denote outliers. *p* values were calculated with an independent sample *t*-test. All *p* values were two-sided with a significance level of 0.05. * *p* < 0.05, ** *p* < 0.01, *** *p* < 0.001.

### 2.4. Electron Microscopy and Evaluation of Mitochondria Morphology

Cells were seeded in 6-well plates at a density of 90,000 cells/well and left to adhere overnight. The following day, cells were fixed in 2.5% glutaraldehyde in 0.1 M sodium cacodylate buffer pH 7.4 (Electron Microscopy Sciences, Hatfield, PA, USA, 16537-15) for 1 h at room temperature, washed three times with 0.2 M sodium cacodylate buffer (Electron Microscopy Sciences, 11650), and fixed in 1% osmium tetroxide, 1.5% potassium ferrocyanide in 0.1 M cacodylate for 1 h on ice. After several washes in distilled water, samples were “en bloc” stained with 0.5% uranyl acetate in water overnight at 4 °C. Finally, samples were dehydrated in a graded ethanol series (30%, 50%,70%, 80%, 90%, 96%, 5 min each, and 3 washes with absolute ethanol, 10 min each). Samples were then infiltrated in a 1:1 ethanol/ Epon 812 solution for 2 h, in 100% Epon twice for 1 h each; then the monolayer cells were covered with a layer of Epon and polymerized in an oven at 60 °C for 48 h. At the end of the polymerization, the plastic was separated from the Epon block by mechanically breaking the plastic, leaving the cell monolayer facing up on the resin block. A portion of the specimen was glued on top of an Epon block and mounted on a Leica Ultracut UCT ultramicrotome. Ultrathin (70–90 nm) sections were then collected on copper grids and stained with uranyl acetate and lead citrate. Grids were examined with a transmission electron microscope (TALOS L120C ThermoScientific, Waltham, MA, USA) at 120 kV. Mitochondria were segmented manually from the EM images. For each mitochondrion mask of every image, size and shape properties were measured with the eccentricity and maximum Feret diameter functions on Matlab.

### 2.5. Autophagy Treatments in Fibroblasts and Cell Lysis

Confluent fibroblasts were treated in the following conditions: 100 nM Torin (Cell Signaling Technology, Danvers, MA, USA, 14379S) for 3 h at 37 °C, 100 nM Bafilomycin A_1_ (Santa Cruz Biotechnology, Dallas, TX, USA, sc-201550) for 3 h at 37 °C, 100 nM Torin and 100 nM Bafilomycin for 3 h at 37 °C, 20 µM carbonyl cyanide *m*-chlorophenyl hydrazine (CCCP) (Sigma, C2759) for 24 h at 37 °C, and 20 µM CCCP and 100 nM Bafilomycin for 24 and 3 h, respectively, at 37 °C. Before each treatment fibroblasts were washed with PBS.

Afterward, fibroblasts were washed with PBS, detached using trypsin (Life Technologies, 25300054), and centrifuged at 500× *g* for 3 min and 30 s. Cell pellets were resuspended in PBS and centrifuged at 1000× *g* for 5 min at 4 °C. Washed pellets were lysed in RIPA buffer (50 mM Tris-HCl, 150 mM NaCl, 1% (*v*/*v*) NP-40, 0.1% (*w*/*v*) SDS, 0.5% (*w*/*v*) deoxycholate, pH 8.0) with freshly added protease inhibitors (Merck, 535140), and incubated for 1 h at 4 °C while constantly spinning. Cell lysates were centrifuged at 15,000× *g* for 15 min at 4 °C, and supernatants were transferred to clean tubes. Protein concentration was determined with a Bradford assay.

### 2.6. Protein Extraction from Tissue Samples

Autoptic or bioptic frozen human tissues were homogenized in 15 volumes of RIPA buffer (Tris-HCl 50 mM pH 7.5, NaCl 150 mM, EDTA 5 mM, NP40 1%, SDS 0.1%, and sodium deoxycholate 0.5%) in the presence of complete protease inhibitors (Roche, 11697498001) using a Dounce homogenizer. Homogenates were incubated on ice for 30 min, cleared by centrifugation at 10,000× *g* at 4 °C for 10 min, and then the protein concentration was determined by Bio-Rad protein assay dye reagent (Bio-Rad, 5000006).

### 2.7. Sample Preparation, SDS-PAGE, and Western Blot

Protein samples were reduced using 4× Laemmli buffer (8% SDS, 40% Glycerol, 0.02% Bromophenol blue, 250 mM Tris-HCl pH 6.8, 0.1 M DTT) and heated at 50 °C for 10 min. Separation of proteins occurred via SDS-PAGE. Samples were loaded on polyacrylamide gels (Bio-Rad, 4561046; or produced *in-house*) and transferred on a PVDF membrane (Bio-Rad, 1704156). After blotting, membranes were washed 3 times with TBS-T (20 mM Tris-HCl, 150 mM NaCl, 1% Tween20) and blocked for 5 min using EveryBlot buffer (Bio-Rad, 12010020). Membranes were incubated with primary antibodies overnight at 4 °C and with secondary antibodies or primary antibodies against housekeeping genes for 1 h at room temperature. Membranes were washed three times with TBS-T between each incubation and before detection. Each antibody was diluted in EveryBlot buffer at the following dilutions: anti-LC3 (Sigma, L7543; 1:20,000), anti-p62 (Progen, Heidelberg, Germany, GP62-C; 1:1000), anti-C19orf12 (produced *in-house*; 1:2000), anti-SDHA (Abcam, ab14715, 1:1000), anti-actin (Santa Cruz, ac-15; 1:50,000), anti-tubulin (Merck, T5168, 1:20,000), anti-rabbit (BioLegend, San Diego, CA, USA, 406401; 1:1000), anti-mouse (Jackson Immuno Research, 115-036-062; 1:10,000), and anti-guinea pig (Abcam, ab130746; 1:1000). Immunological detection was performed using an ECL solution (GE Healthcare, Chicago, IL, USA, RPN2236) and images acquired at Fusion FX (Vilber) instrument. Quantification was performed using Fiji software v. 2.3.0/1.53f [18].

### 2.8. Evaluation of Autophagy in Fibroblasts by Immunocytochemistry

Fibroblasts were seeded on a Permanox slide (Thermo Fisher Scientific, Waltham, MA, USA, 177402) at 7000 cells/well density and left to adhere. Subsequently, cells were treated with 20 μM CCCP for 3, 6, or 24 h and fixed with 4% PFA. Cells were permeabilized using a 0.1% NP-40 solution in PBS, and primary and secondary antibodies were diluted in a 2% BSA 0.1% NP-40 solution in PBS at a 1:200 and 1:500 dilution, respectively. LC3 was immuno-stained with the corresponding antibody (LC3, Sigma, L7543) and detected with a fluorescent-labeled anti-rabbit secondary antibody (Thermo Fisher Scientific, A11034). Nuclei were stained with DAPI (Thermo Fisher Scientific, P36931), and images were acquired by confocal microscopy (Leica TCS SP5). The quantification of LC3 puncta total fluorescence was performed using the Fiji software v. 2.3.0/1.53f using the formula integrated density/area of cell × average mean of background fluorescence to calculate the corrected total cell fluorescence (CTCF).

### 2.9. Screening of Compounds in Fibroblasts by Immunocytochemistry

Fibroblasts were seeded as described in the section above. Cells were simultaneously treated for 24 h with 20 µM CCCP and an autophagy-modulating compound (see Table S2 for the list of compounds and working concentration). Fixing, staining, and image processing were performed as previously described.

## 3. Results

### 3.1. Intracellular Calcium Is Not Consistently Increased in MPAN Fibroblasts

Calcium is an important second messenger that regulates cellular processes such as contraction, proliferation, neurotransmission, and exocytosis. Its homeostasis is guaranteed through the controlled interconnection between plasma membrane channels and intracellular calcium stores such as ER and mitochondria. Based on previous literature, reporting increased mitochondrial calcium in C19orf12^G58S/G58S^ fibroblasts [10], we screened our cohort of primary skin fibroblasts (Table S1 lists cell IDs and corresponding protein variations in C19orf12) with the fluorogenic calcium-sensitive dye Cal-520AM. We measured intracellular calcium in standard culture conditions and after stimulation with the agonist ATP, which induces the release of calcium from intracellular stores.

**Figure 1:**
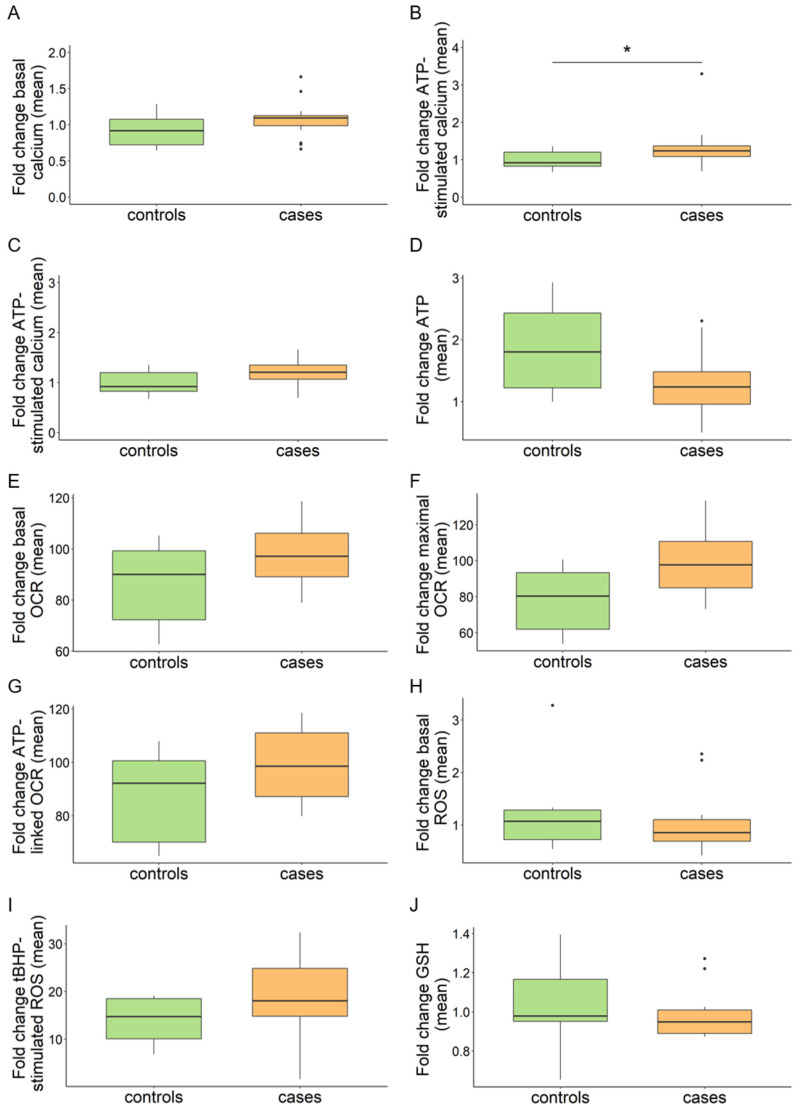
Aggregated values of multi-well plate-based analyses in MPAN (cases) and control fibroblasts (controls). Intracellular calcium (**A**–**C**). (**A**,**B**) Box plots refer to relative fluorescence averages from n = 18 patients (n = 16 AR MPAN; n = 2 AD MPAN) and n = 8 controls (n = 7 carriers of AR MPAN; n = 1, commercial healthy fibroblast line NHDF). (**C**) Results after excluding line 117912 from the patient group. Data refer to standard cell growth conditions (**A**) and stimulation with ATP (**B**,**C**). Total cellular ATP (**D**). Relative luminescence averages from n = 15 patients (n = 13 AR MPAN; n = 2 AD MPAN) and n = 7 controls (n = 6 carriers of AR MPAN; n = 1, commercial healthy fibroblast line NHDF). Mitochondrial bioenergetics by Seahorse (**E**–**G**). Relative OCR averages from n = 10 patients (n = 8 AR MPAN; n = 2 AD MPAN) and n = 6 controls (n = 5 carriers of AR MPAN; n = 1, commercial healthy fibroblast line NHDF). Data refer to standard cell growth conditions (**E**), stimulation with CCCP (**F**), and ATP-linked respiration (**G**). Intracellular ROS (**H**,**I**). Relative DCFDA fluorescence averages from n = 18 patients (n = 16 AR MPAN; n = 2 AD MPAN) and n = 8 controls (n = 7 carriers of AR MPAN; n = 1, commercial healthy fibroblast line NHDF) under standard cell growth conditions (**H**) and n = 14 patients (n = 12 AR MPAN; n = 2 AD MPAN) and n = 4 controls (n = 3 carriers of AR MPAN; n = 1, commercial healthy fibroblast line NHDF) after stimulation with tBHP (**I**). GSH (**J**). Relative GSH fluorescence averages from n = 18 patients (n = 16 AR MPAN; n = 2 AD MPAN) and n = 8 controls (n = 7 carriers of AR MPAN; n = 1, commercial healthy fibroblast line NHDF). Numbers of replicates: patients and carriers n = 10–40; NHDF n = 50. *p* values were calculated with an independent sample *t*-test. All *p* values were two-sided with a significance level of 0.05. * *p* < 0.05.

In our experimental conditions, ATP stimulation led to a 15% increment in intracellular calcium in all cell lines (median NHDF 0.994 vs. 1.147, basal vs. ATP-stimulated). Data were aggregated into the categories of patients and controls. No significant alterations emerged in the patient group under basal conditions, while a significant difference was detected after treating cells with ATP (Figure 1A,B). However, plotting individual cell line values made it apparent that line 117912 with C19orf12^G58S/G58S^ mutations presented a significant increase in intracellular calcium upon ATP stimulation, in line with a previous finding [10]. This was not observed in the other MPAN fibroblasts (Figure S1A,B). After excluding this line from the merged data, the difference between controls and patients became insignificant (Figure 1C). Given that the G58S change falls within the predicted C19orf12 transmembrane domain [2], we searched for additional cell lines with a mutation in this region. We found three siblings (106298, 106299, 106300) with C19orf12^G65V/G65V^ mutations, however, their calcium levels were in the control range. We therefore concluded that there is no general defect in calcium handling in MPAN cells, and that the observed increase in the C19orf12^G58S/G58S^ line is very likely the consequence of a specific effect of the G58S mutation or of the cell lines genetic background, which could include, for instance, additional variants in calcium-related genes.

### 3.2. MPAN Fibroblasts Do Not Show Mitochondrial Bioenergetic and Morphological Alterations

ATP is a critical metabolite and provides energy in various cellular processes. Cells produce ATP through oxidative phosphorylation in mitochondria and glycolysis in the cytosol. Based on a recent report indicating reduced ATP in C19orf12 knock-out (KO) M17 cells in addition to C19orf12^G58S/G58S^ and C19orf12^p.99fs*102^ fibroblasts [14], we evaluated total cellular ATP as a readout of the mitochondrial and cytosolic ATP production, as well as the mitochondrial contribution alone. For total ATP, we screened our fibroblast cohort with a luciferase-based assay. Regarding the calcium measurement, data were aggregated in the categories of patients and controls. No significant alterations emerged in the patient group (Figure 1D) nor in individual MPAN cell lines (Figure S2).

Mitochondrial contribution to respiration and cellular ATP production was assayed with the Seahorse assay in a subset of MPAN fibroblasts. We evaluated oxygen consumption rate (OCR) at the basal and the maximal respiration state, i.e., after stimulation with carbonyl cyanide-4 (trifluoromethoxy) phenylhydrazone (FCCP), and the ATP-linked respiration. No significant alterations were detected in the patient group (Figure 1E–G) or individual MPAN lines in any measured parameter (Figure S3A–C).

Finally, we investigated mitochondrial morphology by electron microscopy. As electron microscopy is not a high throughput technique, we limited our investigations to a smaller number of fibroblast lines. Aiming to cover mutations hitting different protein positions, we selected C19orf12^T11M/G69Rfs*10^, C19orf12^D18G/L132Q^, and C19orf12 ^G69Rfs*10/G69Rfs*10^ fibroblasts for investigation (Figure S4A–D). No defects were detected in the length and eccentricity of the patients’ mitochondria, in contrast to what was concluded in a previous study employing C19orf12^G58S/G58S^ fibroblasts and C19orf12 KO M17 cells [14]. Although we do not have a clear explanation for these discrepant results, based on our extensive analysis of MPAN fibroblasts, we conclude that there is no generalized functional and morphological alteration of mitochondria in MPAN fibroblasts.

### 3.3. Redox Homeostasis Is Not Perturbed in MPAN Fibroblasts

Previous studies report C19orf12^G58S/G58S^ fibroblasts to be more susceptible to oxidative stress-induced cell death [10] and that C19orf12 KO M17 cells, as well as C19orf12^G58S/G58S^ and C19orf12^p.L99fs*102^ fibroblasts, accumulate cytosolic and lipid ROS [14]. As different readouts were used in the two published studies, we systematically quantified total intracellular ROS in our fibroblast cohort using the fluorogenic dye DCFDA under standard conditions and after induction of oxidative stress with tert-Butyl hydroperoxide (tBHP). tBHP stimulation led to a ca. 16-fold increase in intracellular ROS in all cell lines (median NHDF 1.01 vs. 17.04 basal vs. tBHP-stimulated) in our experimental conditions (Figure 1H,I).

We could not detect a consistent ROS increase in the patient group (Figure 1H) and individual MPAN lines (Figure S5A) in standard growth conditions. However, upon tBHP treatment, the median ROS levels of the patient group increased (Figure 1I), albeit not significantly, and the values of individual cell lines tended to cluster toward the left side of the x-axis (Figure S5B), suggesting a trend toward higher ROS production.

To broaden our understanding and to elucidate the status of redox homeostasis in MPAN fibroblasts, we measured reduced glutathione (GSH) using Thiol Green as a proxy of the endogenous oxidative stress defense. No significant decrease of GSH was identified in patient cells (Figure 1J and Figure S5C), supporting the absence of generalized oxidative stress in MPAN fibroblasts.

### 3.4. MPAN Fibroblasts Show Reduced Levels of Autophagosome Formation

**Figure 2:**
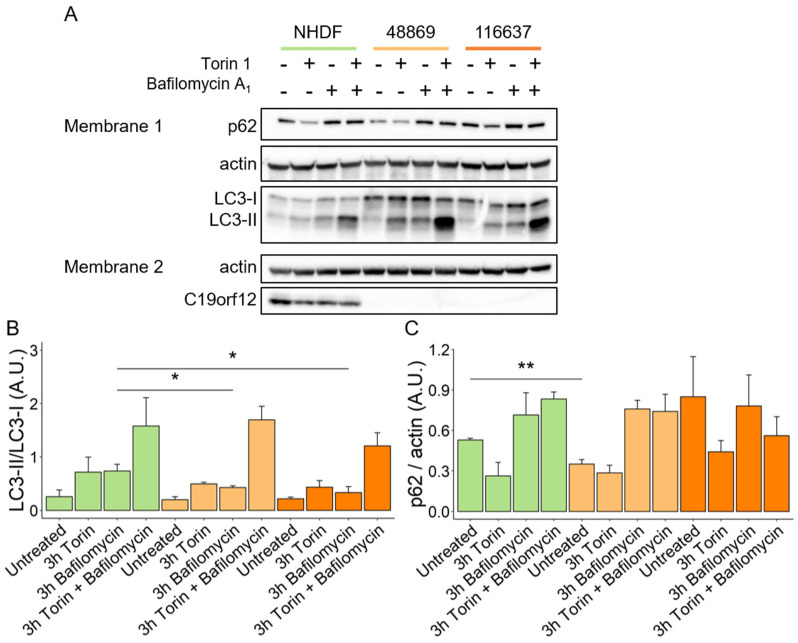
Autophagy flux in MPAN fibroblasts. Representative Western blot analysis of protein expression of LC3-I, LC3-II, p62, actin, and C19orf12 in control (NHDF), C19orf12^D18G/L132Q^ (48869) and C19orf12^G69Rfs*10/G69Rfs*10^ (116637) fibroblasts upon treatment with 100 nM Torin, 100 nM Bafilomycin, and with 100 nM Torin plus 100 nM Bafilomycin Actin was used as a loading control. An amount of 16 μg of cellular lysate proteins were loaded in Membrane 1, and 10 µg in Membrane 2 (**A**). Relative abundance of LC3-II versus LC3-I (**B**). Quantification of p62 expression level relative to actin (**C**). Chemiluminescent protein signals were quantified using Fiji v. 2.3.0/1.53f n = 3 independent experiments were considered for the quantification. Data were analyzed with unpaired two-tailed *t*-test; * *p* < 0.05, ** *p* < 0.01.

HeLa cells overexpressing mutant G58S and Q96P C19orf12 proteins showed inefficient conversion of the autophagy marker LC3-I into the active form LC3-II upon induction of bulk autophagy by starvation [10]. To confirm these data and provide insight into autophagy initiation and flux in MPAN, we investigated autophagosome formation in transgene-free patient fibroblasts. Using a Western blotting approach, we quantified the expression of p62 and the LC3-I/II ratio in patients’ fibroblasts after treatments with Torin 1, an autophagy inducer, Bafilomycin A_1_, an autophagy inhibitor, and the combination of both (Figure 2A, membrane 1). After treatment with Bafilomycin A_1_, MPAN cells showed reduced conversion of LC3-I to LC3-II, and a similar trend was present following Torin 1 treatment, implying impairment of basal autophagy in MPAN (Figure 2B). Normal levels of p62 excluded the possibility of autophagy flux blockage in MPAN fibroblasts (Figure 2C).

### 3.5. MPAN Fibroblasts Show Reduced Amount of LC3 Puncta That Can Be Rescued by Selective Compounds

**Figure 3:**
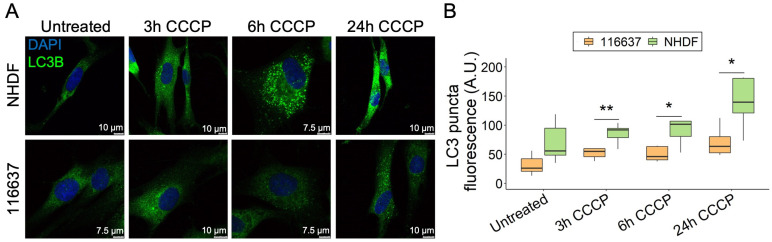
Time-course of LC3 puncta formation in C19orf12^G69Rfs*10/G69Rfs*10^ fibroblasts treated with CCCP. Representative images of C19orf12^G69Rfs*10/G69Rfs*10^ (116637) and control NHDF fibroblasts treated for 3, 6, and 24 h with 20 µM CCCP. The green signal indicates LC3-II puncta; the blue signal (DAPI), nuclei (**A**). Quantification of LC3 puncta fluorescence was performed using ImageJ. n = 10 cells from each of the n = 2 independent experiments were considered for the quantification. *p* values were calculated with an independent sample *t*-test. All *p* values were two-sided with a significance level of 0.05. * *p* < 0.05, ** *p* < 0.01 (**B**).

Due to the mitochondrial localization of C19orf12, we investigated the possibility that C19orf12 is involved in mitophagy, i.e., the specialized form of autophagy that selectively removes dysfunctional mitochondria, besides bulk autophagy. Using the protonophore carbonyl cyanide *m*-chlorophenyl hydrazine (CCCP), we depolarized mitochondria of C19orf12^G69Rfs*10/G69Rfs*10^ fibroblasts and tracked LC3 puncta fluorescence by confocal microscopy at different time points (Figure 3A). At 3, 6, and 24 h from induction, LC3 puncta were increased both in the control and patient fibroblasts, although at each time point, the number of LC3 puncta was significantly lower in patient cells compared with control cells, with the most significant difference at 24 h (Figure 3B).

**Figure 4:**
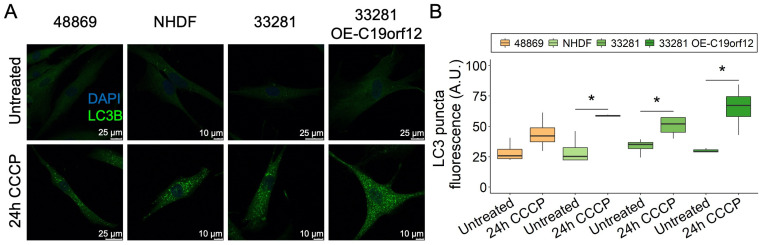
LC3 puncta formation in C19orf12^D18G/L132Q^ fibroblasts after 24 h of CCCP treatment. Representative images of C19orf12^D18G/L132Q^ fibroblasts (48869) and control cells (NHDF, 33281) and cells over-expressing C19orf12 (33281-T-C19orf12) treated for 24 h with 20 µM CCCP. The green signal indicates LC3 puncta; the blue signal (DAPI), nuclei (**A**). Quantification of LC3-II fluorescence was performed using ImageJ. n = 10 cells from each of the n = 4 independent experiments were considered for the quantification. Data were analyzed with paired one-tailed *t*-tests; * *p* < 0.05 (**B**).

We extended the analysis to C19orf12^D18G/L132Q^ fibroblasts and two additional controls, including a fibroblast line overexpressing wild-type C19orf12 (Figure 4). After 24 h of CCCP treatment, LC3 puncta increased in all cell lines, but only in control fibroblasts was the increase significant (Figure 4B). Of note, control cells overexpressing wild-type C19orf12 had the highest amount of LC3 puncta, suggesting that autophagy activation positively correlates with the expression of functional *C19orf12* alleles (Figure 4B). Altogether, immunofluorescence data indicating fewer LC3 puncta in MPAN cells and Western blotting data showing reduced LC3-II/I ratio suggest that MPAN cells generate fewer autophagosomes compared with control cells. However, despite the reduction of LC3 puncta in MPAN fibroblasts treated with CCCP compared with control cells, there was no significant difference in the overall clearance of mitochondria (Figure S6A,B), suggesting that in dividing cells, C19orf12 contributes to the early regulation of autophagy, but that it is redundant for mitochondrial removal/mitophagy and autophagic flux.

**Figure 5:**
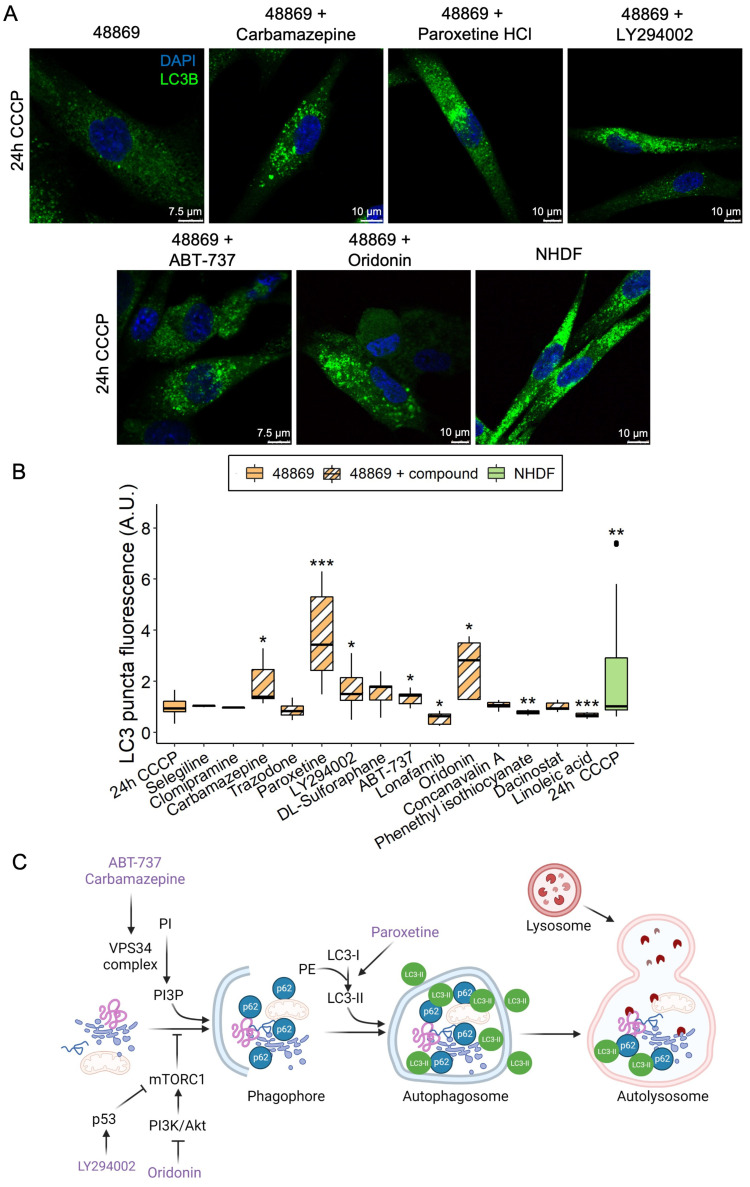
LC3 puncta levels in C19orf12^D18G/L132Q^ fibroblasts treated with autophagy-modulating compounds. Representative confocal images of C19orf12^D18G/L132Q^ fibroblasts treated for 24 h with 20 µM CCCP only and with CCCP plus each of the five hit compounds (carbamazepine, paroxetine, LY294002, ABT-737, and oridonin). The green signal indicates LC3; the blue signal (DAPI), nuclei (**A**). Box plots refer to LC3 puncta fluorescence levels normalized per number of cells in NHDF and C19orf12^D18G/L132Q^ fibroblasts (48869) treated with 20 µM CCCP alone and C19orf12^D18G/L132Q^ fibroblasts treated with CCCP plus autophagy modulating compounds. Quantification of LC3 puncta fluorescence was performed using ImageJ. n = 10 cells from each of the n = 6 independent experiments were considered for the quantification. Data were analyzed with unpaired two-tailed *t*-tests; * *p* < 0.05, ** *p* < 0.01, *** *p* < 0.001 (**B**). Schematic representation of the entry point of each hit compound in the autophagy flux. The picture was created using BioRender (**C**).

Based on the reduced amount of LC3 puncta in MPAN fibroblasts, we screened 14 compounds known from the literature to modulate autophagy (Table S2) in C19orf12^D18G/L132Q^ fibroblasts. Cells were either treated with CCCP alone or simultaneously with CCCP and an autophagy-modulating compound for 24 h, and LC3 puncta were subsequently measured (Figure 5A,B).

**Figure 6:**
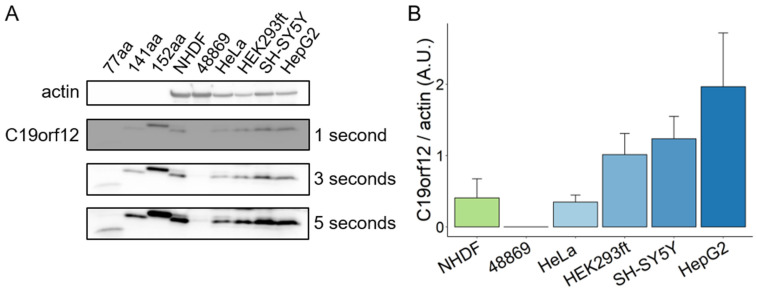
C19orf12 expression in human cells. Representative Western blot analysis of C19orf12 expression in cellular lysates. Recombinant C19orf12 proteins (77, 141, 152 aa) were used as molecular weight references besides the protein ladder. Actin was used as a loading control. An amount of 10 μg of cellular lysate proteins and 1ng of recombinant proteins were loaded (**A**). Relative quantification of C19orf12 (141 aa) expression to actin. Chemiluminescent protein signals were quantified under no-saturating conditions (three seconds exposure time) using ImageJ (**B**). n = 4 independent experiments were considered for the quantification.

Phenethyl isothiocyanate was toxic to the cells, causing massive cell death, and those cells still alive after treatment demonstrated reduced LC3 puncta levels. Lonafarnib and linoleic acid reduced LC3 puncta levels without being toxic to the cells. Clomipramine, trazodone, sulforaphane, dacinostat, selegiline, and concanavalin A did not affect cell viability and LC3 puncta levels. In contrast, carbamazepine, paroxetine, LY294002, ABT-737, and oridonin rescued LC3 puncta in patient cells to a level comparable to or higher than control fibroblasts (Figure 5B). Despite belonging to different classes of compounds, the five hits seem to all be involved in the initiation of the autophagy process (Figure 5C). The barely detectable C19orf12 protein in the C19orf12^D18G/L132Q^ cell line (see Figure 2A and Figure 6 for comparison of C19orf12’s immunological signal at different exposure and saturation conditions) suggests that these mutations cause a significant effect on the protein stability. It is, therefore, likely that the five identified compounds act through a C19orf12-independent mechanism to enhance autophagy. This implies that the autophagy-restoring effect of these compounds works in any MPAN cell, regardless of the mutations and residual C19orf12 levels in the cell.

### 3.6. Fibroblasts Are a Valid Tool for Modeling MPAN

*C19orf12* is highly expressed in adipose tissue, brain, and muscle, while its expression is substantially reduced in cultured fibroblasts [11]. This opens the question of how relevant fibroblasts are to model MPAN. Using a C19orf12-specific antibody capable of detecting the main C19orf12 isoforms (UniProt Q9NSK7-1, 152 amino acids (aa) residues; Q9NSK7-4, 141 aa; Q9NSK7-2, 77 aa), we performed Western blotting on lysates of various human cell types (fibroblasts, cervical, kidney, hepatic, and neuronal cells) and tissues (heart, kidney, muscle, liver, brain, and adipose) to identify tissue-specific isoform expression and relative abundance. C19orf12^D18G/L132Q^ fibroblasts were used as the negative control, and recombinant C19orf12 proteins of 152, 141, and 77 aa residues as molecular weight references.

The results show that C19orf12 is consistently detected in all investigated cell types and tissues, and is barely detectable in C19orf12^D18G/L132Q^ patient cells (Figure 6A,B). This suggests that there are no tissue-specific isoforms, and that C19orf12 might be involved in a basic common mechanism. Furthermore, we showed that the 141 aa isoform is the prevalently expressed C19orf12 isoform in all cell types, suggesting this to be the canonical form, contrary to previous knowledge indicating the 152 aa isoform to be canonical [2,10]. A faint band corresponding to a 152 aa protein could be detected in control fibroblasts, HeLa, and SH-SY5Y cells at longer exposure times (Figure 6A). We could not see a band corresponding to a 77 aa isoform in any of the investigated cell types and tissues, to the detection sensitivity of the Western blotting technique (Figure 6 and Figure S7).

## 4. Discussion

In this study, we employed a large number of MPAN fibroblasts from patients with AR and AD forms and controls to identify phenotypes reliably shared in MPAN for testing pharmacological treatments. We focused our investigations on mitochondria-related phenotypes given the subcellular localization of C19orf12. Fibroblasts from carriers of AR MPAN were used as controls to match the genetic background of the patients to the best extent possible. Because of the large size of our sample cohort, we used high-throughput assays to investigate parameters whenever multi-well-based approaches were available. Investigations requiring confocal microscopy, electron microscopy, and Western blotting were instead performed on a smaller number of samples due to the limited throughput intrinsic to the methods.

Under the tested experimental conditions, we found no evidence of mitochondrial dysfunction in MPAN fibroblasts, as the investigated parameters (calcium handling, mitochondrial bioenergetics and morphology, ROS generation, and antioxidant defense) were not consistently impaired in all patients. We did not explore iron and ferritin levels, however, the absence of increased ROS, and normal GSH levels, spoke against eliciting ferroptosis in MPAN fibroblasts.

The absence of a consistent mitochondrial impairment in MPAN fibroblasts in our study is probably due to the fact that we used cells from multiple individuals, thus increasing interindividual variability and producing data with larger variation, when compared with studies that used repeated measures in smaller data sets, as in [14]. While our approach likely neglects subtle defects, it has the advantage of identifying robust phenotypes shared by all MPAN fibroblasts.

A significant increase in intracellular calcium was identified only in the cell line with C19orf12^G58S/G58S^ mutations after ATP stimulation. A similar result was reported in a previous study [10] using fibroblasts from the same donor, assessed with a different approach (fluorogenic dye Cal520 for total cellular calcium in the current study vs. aequorin for mitochondria-associated calcium in Venco et al. [10]). We exclude the association of elevated calcium level with the localization of the G58S mutation in a specific C19orf12 domain, in this case the predicted transmembrane domain, as an additional mutation also clustering in the transmembrane domain (G65V), was not accompanied by high intracellular calcium levels. We therefore speculate that the elevated calcium level in this isolated cell line stems from either a specific effect of the G58S mutation or additional variants in genes relevant to calcium homeostasis. Of note, C19orf12^G58S/G58S^ fibroblasts in the current and two previous studies [10,14] were retrieved from the Telethon Network of Genetic Biobanks, indicating that fibroblasts from the same donor were employed in all studies. Future studies in fibroblasts from a different donor carrying the same variant and trio sequencing in patient 117912 and carriers will help clarify this aspect.

In the current study, we also point out that the effects of AD MPAN mutations on mitochondrial cellular phenotypes are indistinguishable from those of AR mutations, providing evidence that the overlap of clinical and pathology aspects in AD and AR MPAN patients [3] is also substantiated by overlap at a functional level.

By investigating autophagy in naïve MPAN fibroblasts, we corroborated the finding that C19orf12 intervenes in the initiation of the autophagy process by supporting the conversion of LC3-I to LC3-II without binding directly to LC3 [10] and, possibly, by promoting the maintenance of a pool of functioning mitochondria. Although the accumulation of dysfunctional mitochondria upon CCCP treatment did not reach the threshold for statistical significance in MPAN fibroblasts, results in neurons could be different. Fibroblasts can dilute damaged mitochondria through cell division, in addition to removing them via autophagy, while neuronal cells, being non-dividing cells, rely mainly on autophagy to clear dysfunctional organelles [19]. Investigating clearance of mitochondria in patient-derived neuronal cells could provide compelling evidence of an additional role in mitophagy for C19orf12, besides a role in initiating autophagy.

As the conversion of LC3-I to LC3-II relies on the conjugation of LC3-I to a phosphatidylethanolamine (PE) moiety, defective lipidation of LC3 in MPAN may point to a primary role for C19orf12 in the conjugation of PE to LC3-I, or in the synthesis of PE between ER and mitochondria. Considering C19orf12 localization at the mitochondria, ER, and ER-mitochondria contact sites, C19orf12 could tether mitochondria and ER to allow efficient transfer of the PE precursor, phosphatidylserine, from ER to mitochondria, its conversion to PE in mitochondria and transfer back to the ER for phosphatidylcholine production. By integrating studies in cellular models with dynamic lipid metabolism and structural biology, we hope to unambiguously clarify the function of C19orf12 soon.

Despite the fact that the primary function of C19orf12 is still to be defined, mutations in *C19orf12* result in impaired activation of LC3. Therefore, any pharmacological treatment restoring LC3 activity could be used as a therapeutic compound for MPAN. To this end, we screened 14 autophagy-modulating compounds in MPAN fibroblasts and identified five that were able to increase the LC3 levels in patient fibroblasts: carbamazepine, LY294002, ABT-737, oridonin, and paroxetine. Of note, LY294002 and paroxetine may also have an inhibitory effect on autophagy [20,21], suggesting a dual effect for these compounds, similarly to DL-Sulforaphane, also tested in our screening [22,23]. Still, the dual effect seems to depend on the cell type tested [24], and the concentration of the compound, as reported for LY294002 [20,25].

Although the five hits take part in various biological processes (anticonvulsant, antidepressant, anti-tumoral, and inhibitors of phosphoinositide 3-kinases and Bcl-2), based on a literature search, they have a shared effect in stimulating the initial steps of the autophagy process. Oridonin and LY294002 inhibit the mechanistic target of rapamycin complex 1 (mTORC1) by inhibiting the PI3K/Akt pathway or activating p53, respectively [25,26]. Carbamazepine and ABT-737 promote the activity of VPS34 complex [27,28]. Paroxetine stimulates LC3-I conversion to LC3-II [29,30,31]. Notably, carbamazepine, oridonin, and paroxetine are molecules approved by the Food and Drug Administration, implying a realistic clinical translatability in MPAN, as opposed to LY294002 and ABT-737, which still require further validation.

Although fibroblasts cannot recapitulate tissue-specific metabolic aspects, conserved processes such as autophagy can be reliably investigated due to the conservation of the autophagy machinery and the prominent expression of the 141 aa isoform in fibroblasts, as well as in all other investigated cell types and tissues. In conclusion, while further research is needed to identify the primary function of C19orf12, our study moves one step closer to understanding the consequences of C19orf12 dysfunction at the cellular level and to establishing a rational therapy for MPAN.

## Data Availability

Data are contained within the article or supplementary material.

## Supplementary Materials

The following supporting information can be downloaded at: https://www.mdpi.com/article/10.3390/pharmaceutics15010267/s1, Table S1: List of primary skin fibroblasts derived from MPAN patients and carriers; Table S2: List of compounds screened in MPAN fibroblasts; Figure S1: Intracellular calcium in individual fibroblasts. Box plots refer to individual cell line values of relative fluorescence. Relative fold changes in patients and controls are normalized to the protein-normalized average fluorescence of NHDF cells in standard growth conditions. Data are presented by decreasing medians before (A) and after ATP stimulation (B); Figure S2: Total cellular ATP in individual fibroblasts; Figure S3: Mitochondrial bioenergetics by Seahorse assay in individual fibroblasts. Box plots refer to individual cell line values of OCR. Relative fold changes in patients and controls are normalized to the cell number-normalized average OCR of NHDF cells. Data are presented by decreasing medians. Data show results of basal (A) and maximal respiration after stimulation with CCCP (B) and ATP-linked respiration (C); Figure S4: Evaluation of the mitochondrial morphology in MPAN fibroblasts. Representative pictures of electron microscopy sections from healthy fibroblast line (NHDF) and C19orf12^T11M/G69Rfs*10^ (46572), C19orf12^D18G/L132Q^ (48869) and C19orf12^G69Rfs*10/G69Rfs*10^ (116636) fibroblasts in Epon resin. Mitochondria are labeled in green (A). Grey masks were used to extract the mitochondrial descriptors (B). Box plots reporting the quantification of mitochondrial length (C) and eccentricity (D) extracted from the mitochondrial masks; Figure S5: Intracellular ROS and GSH levels in individual fibroblasts. ROS (A,B). Box plots refer to individual cell line values of relative fluorescence. Relative ROS fold changes in patients and controls are normalized to the protein-normalized average fluorescence of NHDF cells in standard growth conditions. Data are presented by decreasing medians before (A) and after tBHP stimulation (B). GSH (C); Figure S6: Residual mitochondrial succinate dehydrogenase (SDH) protein level upon CCCP treatment. Representative western blot analysis of protein expression of SDH, subunit A, (SDHA), and tubulin in control (NHDF) C19orf12^D18G/L132Q^ (48869) and C19orf12^G69Rfs*10/G69Rfs*10^ (116637) fibroblasts upon treatment with 20 μM CCCP for 24 h. Actin was used as a loading control. 10 μg proteins of cellular lysates were loaded (A). Actin was used as a loading control. 10 μg proteins of cellular lysates were loaded. Relative abundance of SDHA in cells treated with CCCP vs. untreated cells (B); Figure S7: C19orf12 expression in human tissues. (A) Representative western blot analysis of C19orf12 expression in tissue lysates. Recombinant C19orf12 proteins (77, 141, 152 aa) were used as molecular weight references besides the protein ladder. Tubulin was used as a loading control. The following amounts of proteins were loaded; 60 μg of tissue lysates; 1.5 ng of 77aa; 0.9 ng of 141aa; 0.3 ng of 152 aa. (B) Relative quantification of C19orf12 expression to tubulin.
