# Supplementary material for: Identification of Autophagy as a Functional Target Suitable for the Pharmacological Treatment of Mitochondrial Membrane Protein-Associated Neurodegeneration (MPAN) In Vitro
Source: Pharmaceutics. 2023 Jan 12;15(1):267. doi: 10.3390/pharmaceutics15010267 (PMC9862353; doi:10.3390/pharmaceutics15010267)
Supplement: Supplementary file 1 [file pharmaceutics-15-00267-s001.zip › pharmaceutics-2089522-supplementary.pdf]

Table S1. List of primary skin fibroblasts derived from MPAN patients and carriers. From left to right, columns indicate in order: 1. Numerical ID assigned to each fibroblast cell line; 2. Pedigree ID in case of fibroblasts from patients and carriers belonging to the same family; 3. Relation to the index and form of MPAN: autosomal recessive (AR) or autosomal dominant (AD); 4. Protein variants in C19orf12.

Fibroblast ID	Pedigree	Relationship to index	Protein variant in C19orf12
45966		index (AR)	p.[Ala67Glyfs*16];[Ala67Glyfs*16]
46572		index (AR)	p. [Thr11Met];[Gly69Argfs*10]
48869		index (AR)	p. [Asp18Gly];[Leu132Gln]
106298	NBIA384	index (AR)	p. [Gly65Val];[Gly65Val]
106299		affected relative (AR)	p. [Gly65Val];[Gly65Val]
106300		affected relative (AR)	p. [Gly65Val];[Gly65Val]
118245		unaffected carrier	p. [Gly65Val];[=]
118246		unaffected carrier	p. [Gly65Val];[=]
116351	NBIA515	index (AR)	p.[Gln97Pro];[Gly69Argfs*10]
120563		unaffected carrier	p.[Gln97Pro];[=]
120564		unaffected carrier	p.[Gly69Argfs*10];[=]
116636	NBIA510	index (AR)	p. [Gly69Argfs*10];[Gly69Argfs*10]
118373		unaffected carrier	p. [Gly69Argfs*10];[=]
118374		unaffected carrier	p. [Gly69Argfs*10];[=]
116637		index (AR)	p. [Gly69Argfs*10];[Gly69Argfs*10]
116638		index (AR)	p. [Gly69Argfs*10];[Gly69Argfs*10]
116641		index (AR)	p. [Gly69Argfs*10];[Gly69Argfs*10]
116642		index (AR)	p. [Gly69Argfs*10];[Gly69Argfs*10]
116643		index (AR)	p. [Gly69Argfs*10];[Gly69Argfs*10]
116673		index (AR)	p. [Gly69Argfs*10];[Gly69Argfs*10]
117912		index (AR)	p.[Gly58Ser]; [Gly58Ser]
118375	NBIA#027	index (AR)	p. [Thr11Met];[Gly69Argfs*10]
118376		unaffected carrier	p. [Thr11Met];[=]
118377		unaffected carrier	p.[Gly69Argfs*10];[=]
128054		index (AD)	p.Ala94Serfs*24
128055		index (AD)	p.Met76Thrfs*3
NHDF		Healthy individual	p. [=];[=]
33281		Undiagnosed mitochondrial patient, PMID: 22499348	p. [=];[=]

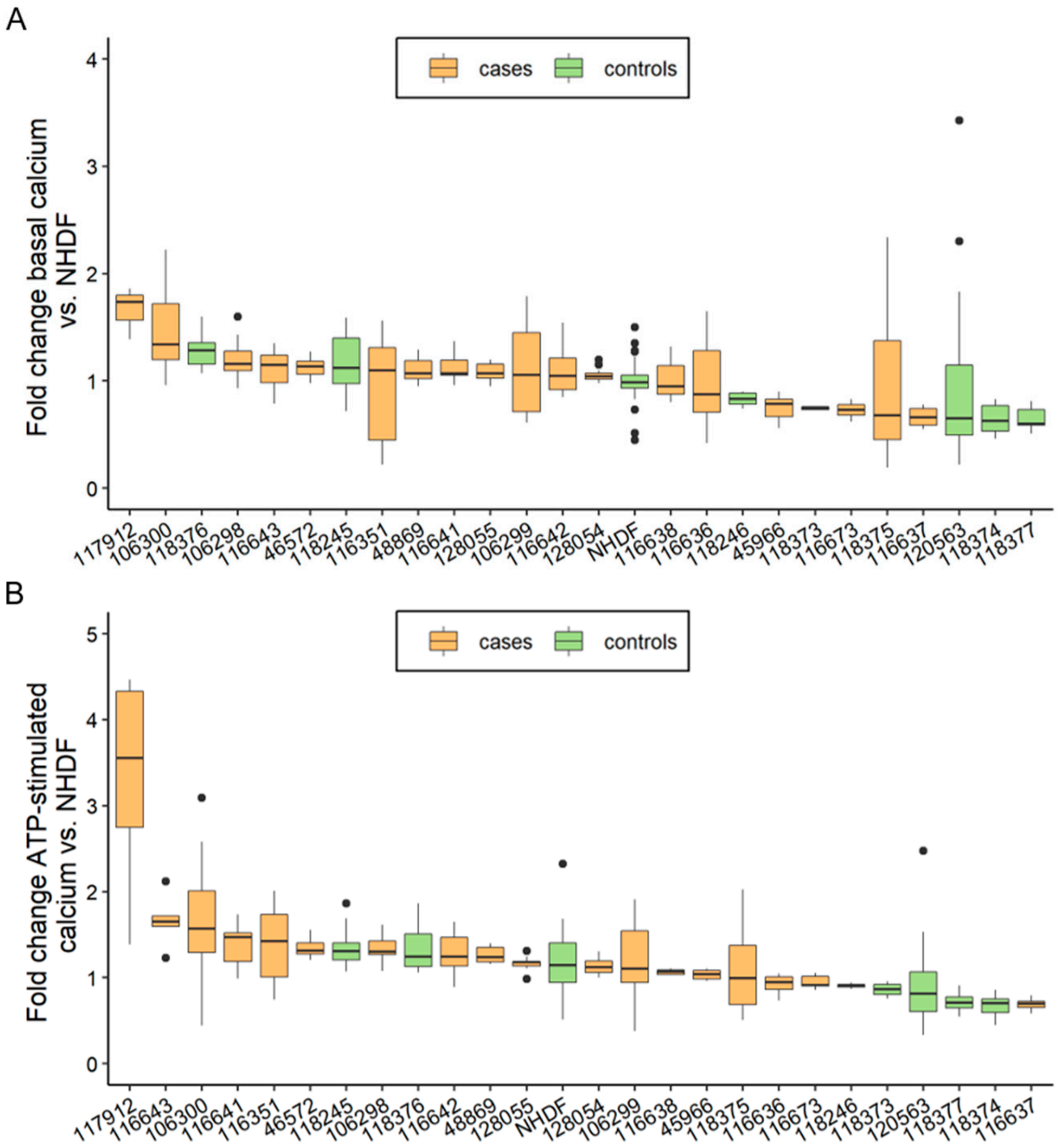

Figure S1. Intracellular calcium in individual fibroblasts. Box plots refer to individual cell line values of relative fluorescence. Relative fold changes in patients and controls are normalized to the protein-normalized average fluorescence of NHDF cells in standard growth conditions. Data are presented by decreasing medians before (A) and after ATP stimulation (B). Numbers of replicates: patients and carriers n=10-40; NHDF n=50. p values were calculated with an independent sample t-test. All p values were two-sided with a significance level of 0.05.

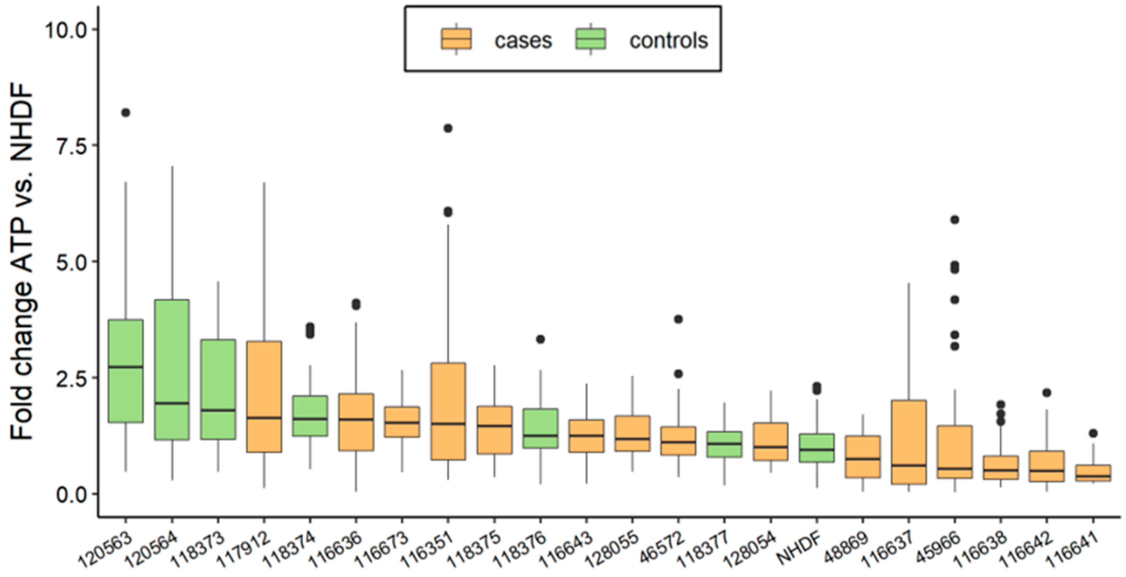

Figure S2. Total cellular ATP in individual fibroblasts. Box plots refer to individual cell line values of relative luminescence. Relative fold changes in patients and controls are normalized to the protein-normalized average luminescence of NHDF cells. Data are presented by decreasing medians. Numbers of replicates: patients and carriers n=10-40; NHDF n=50. p values were calculated with an independent sample t-test. All p values were two-sided with a significance level of 0.05.

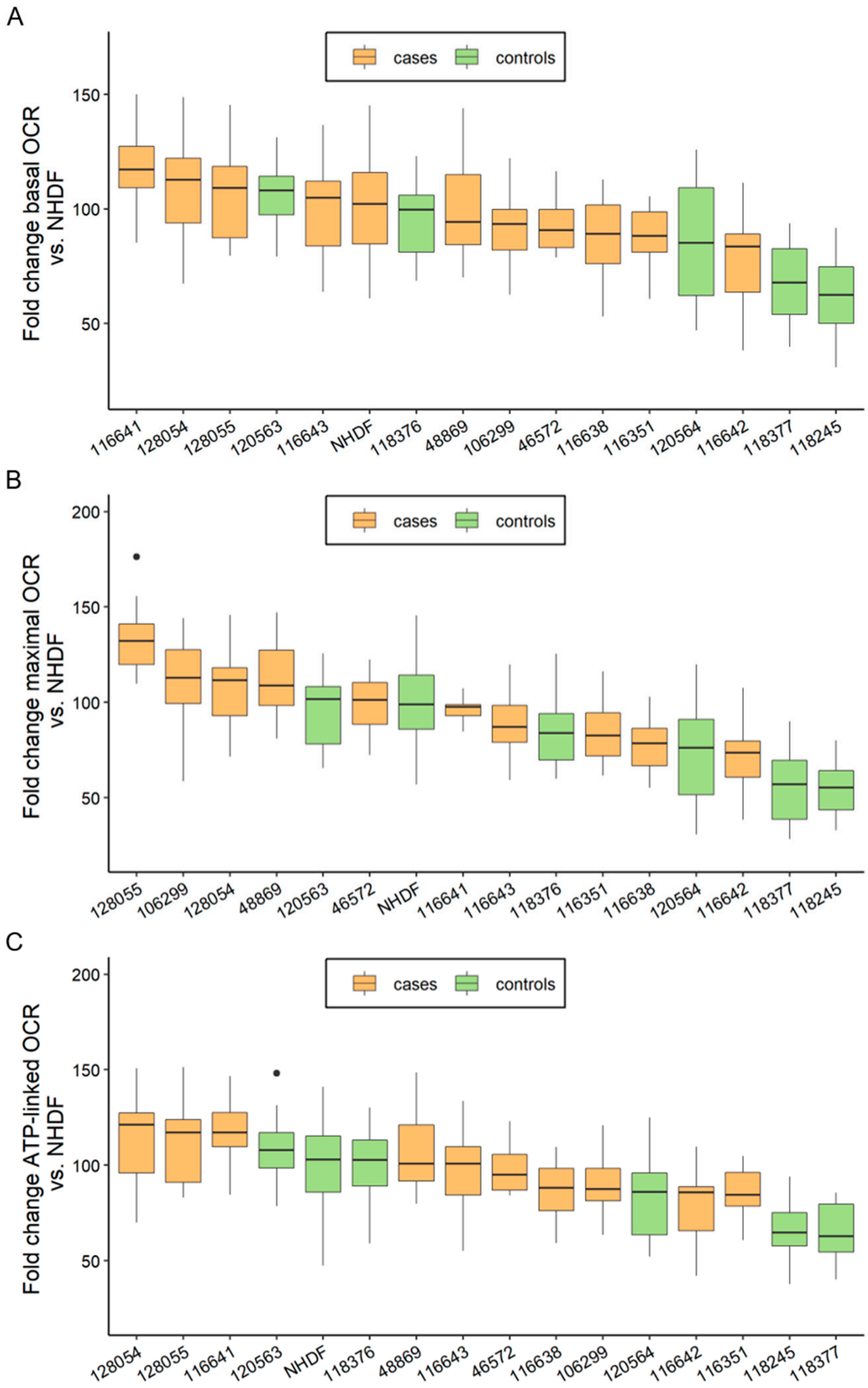
A
cases
controls
Fold change basal OCR vs. NHDF
150
100
50
116641
128054
128055
120563
116643
NHDF
118376
48869
106299
46572
116638
116351
120564
116642
118377
118245
B
cases
controls
Fold change maximal OCR vs. NHDF
200
150
100
50
128055
106299
128054
48869
120563
46572
NHDF
116641
116643
118376
116351
116638
120564
116642
118377
118245
C
cases
controls
Fold change ATP-linked OCR vs. NHDF
200
150
100
50
128054
128055
116641
120563
NHDF
118376
48869
116643
46572
116638
106299
120564
116642
116351
118245
118377

Figure S3. Mitochondrial bioenergetics by Seahorse assay in individual fibroblasts. Box plots refer to individual cell line values of OCR. Relative fold changes in patients and controls are normalized to the cell number-normalized average OCR of NHDF cells. Data are presented by decreasing medians. Data show results of basal (A) and maximal respiration after stimulation with CCCP (B) and ATP-linked respiration (C). Numbers of replicates: patients and carriers n=10-40; NHDF n=50. p values were calculated with an independent sample t-test. All p values were two-sided with a significance level of 0.05.

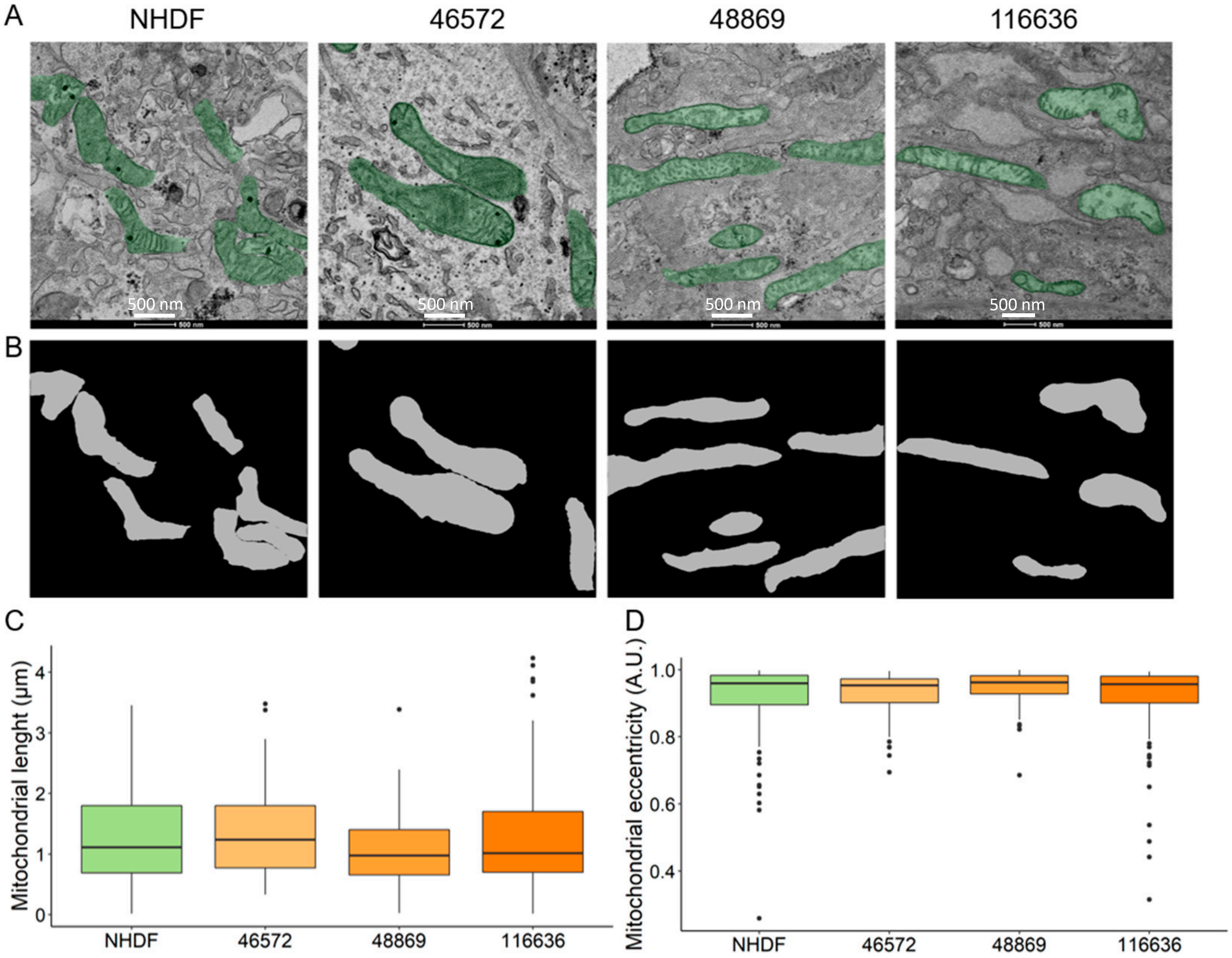

Figure S4. Evaluation of the mitochondrial morphology in MPAN fibroblasts. Representative pictures of electron microscopy sections from healthy fibroblast line (NHDF) and C19orf12$^{T11M/G69Rfs*10}$ (46572), C19orf12$^{D18G/L132Q}$ (48869) and C19orf12$^{G69Rfs*10/G69Rfs*10}$ (116636) fibroblasts in Epon resin. Mitochondria are labeled in green (A). Grey masks were used to extract the mitochondrial descriptors (B). Box plots reporting the quantification of mitochondrial length (C) and eccentricity (D) extracted from the mitochondrial masks. Numbers of replicates: patients n=252; NHDF n=162. p values were calculated with Kruskal-Wallis statistical test. All p values were two-sided with a significance level of 0.05.

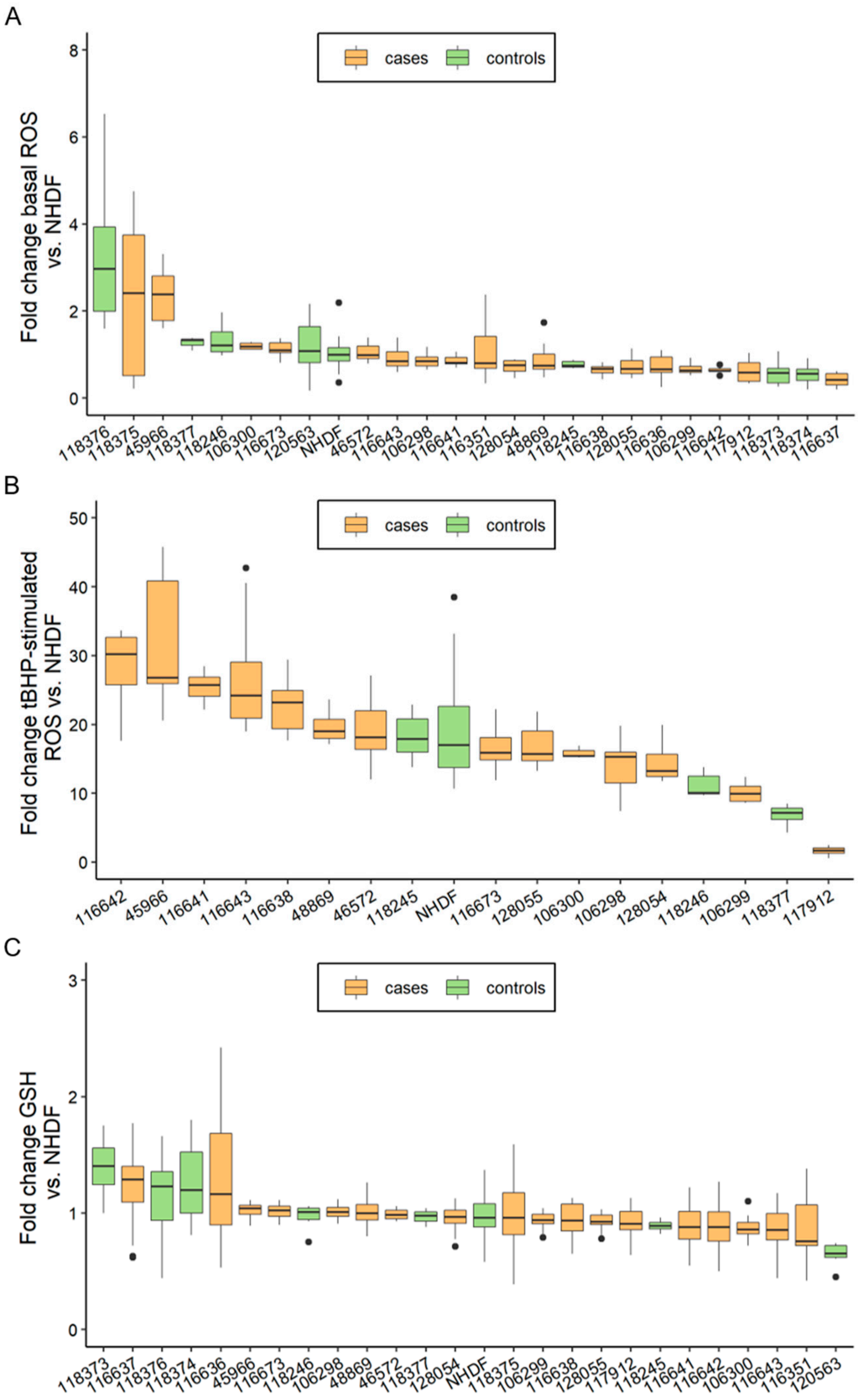
A
cases
controls
Fold change basal ROS vs. NHDF
0
2
4
6
8
118376
118375
45966
118377
118246
106300
116673
120563
NHDF
46572
116643
106298
116641
116351
128054
48869
118245
116638
128055
116636
106299
116642
117912
118373
118374
116637
B
cases
controls
Fold change tBHP-stimulated ROS vs. NHDF
0
10
20
30
40
50
116642
45966
116641
116643
116638
48869
46572
118245
NHDF
116673
128055
106300
106298
128054
118246
106299
118377
117912
C
cases
controls
Fold change GSH vs. NHDF
0
1
2
3
118373
116637
118376
118374
116636
45966
116673
118246
106298
48869
46572
118377
128054
NHDF
118375
106299
116638
128055
117912
118245
116641
116642
106300
116643
116351
120563

Figure S5. Intracellular ROS and GSH levels in individual fibroblasts. ROS (A,B). Box plots refer to individual cell line values of relative fluorescence. Relative ROS fold changes in patients and controls are normalized to the protein-normalized average fluorescence of NHDF cells in standard growth conditions. Data are presented by decreasing medians before (A) and after tBHP stimulation (B). **GSH (C).** Relative GSH fold changes in patients and controls are normalized to the protein-normalized average fluorescence of NHDF cells.

Numbers of replicates: patients and carriers n=10-40; NHDF n=50. p values were calculated with an independent sample t-test. All p values were two-sided with a significance level of 0.05.

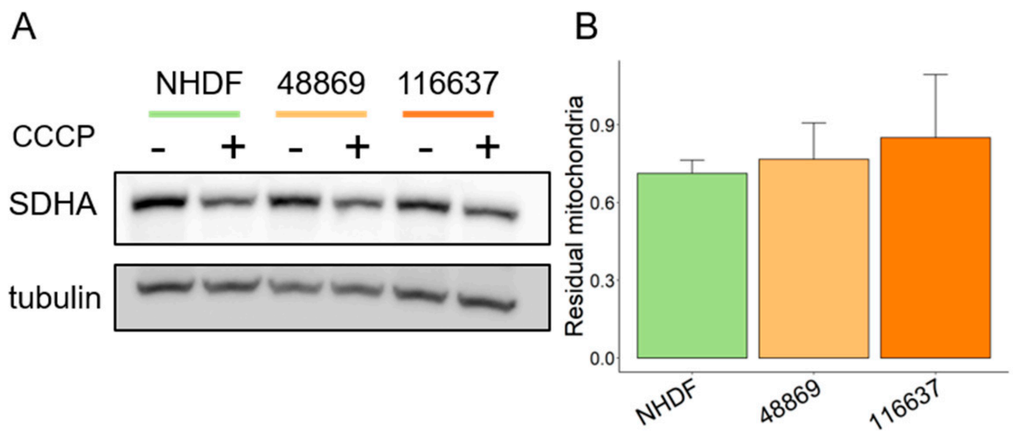

Figure S6. Residual mitochondrial succinate dehydrogenase (SDH) protein level upon CCCP treatment. Representative western blot analysis of protein expression of SDH, sub-unit A, (SDHA), and tubulin in control (NHDF) C19orf12$^{D18G/L132Q}$ (48869) and C19orf12$^{G69Rfs*10/G69Rfs*10}$ (116637) fibroblasts upon treatment with 20 µM CCCP for 24 hours. Actin was used as a loading control. 10 µg proteins of cellular lysates were loaded (A). Actin was used as a loading control. 10 µg proteins of cellular lysates were loaded. Relative abundance of SDHA in cells treated with CCCP vs. untreated cells (B). Chemiluminescent protein signals were quantified using ImageJ. n = 4 independent experiments were considered for the quantification. Data were analyzed with unpaired two-tailed t-test; *$p < 0.05$.

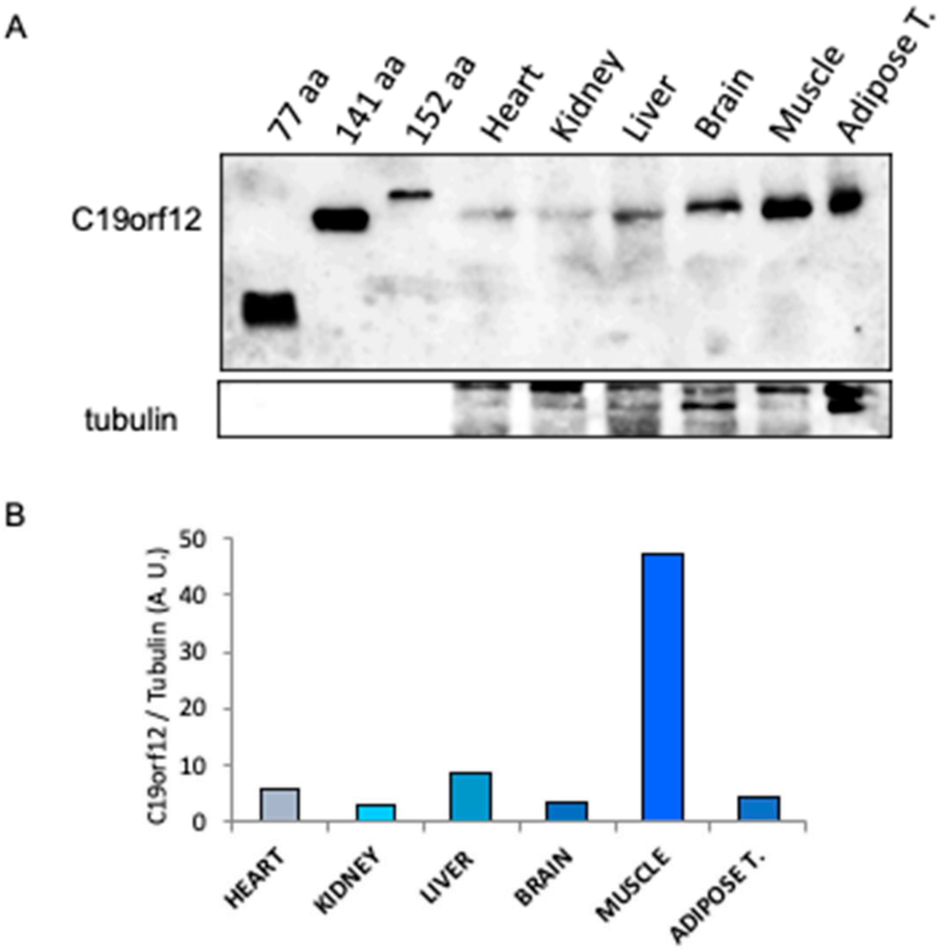

Figure S7. C19orf12 expression in human tissues. (A) Representative western blot analysis of C19orf12 expression in tissue lysates. Recombinant C19orf12 proteins (77, 141, 152 aa) were used as molecular weight references besides the protein ladder. Tubulin was used as a loading control. The following amounts of proteins were loaded; 60 µg of tissue lysates; 1.5 ng of 77aa; 0.9 ng of 141aa; 0.3 ng of 152 aa. (B) Relative quantification of C19orf12 expression to tubulin.

Table S2. List of compounds screened in MPAN fibroblasts. From left to right, columns indicate in order: 1. List of the fourteen compounds used in the current study; 2. SMILES descriptors; 3. Working concetration of each compound in cell culture medium; 4. Effect of the compound on LC3 puncta modulation in the current study; 5. Effect of the compound on LC3 based on the literature 6. Literature references for selected compounds.

Compounds screened	SMILES	Final concentration	Effects on LC3-II levels	Effect on autophagy based on literature	Literature reference (PMID)
Clomipramine	CN(C)CCCN1C2=C(CCC3=C1C=C(Cl)C=C3)C=CC=C2	10µM	-	Inhibition	19706685
Oridonin	CC1(C)CCC(O)[C@]23CO[C@](O)(C(O)[C@H]12)[C@@]12C(O)C(CC[C@@H]31)C(=C)C2=O	2µg/µL	**Increase**	Induction	17895587
Paroxetine	FC1=CC=C(C=C1)C1CCNCC1COC1=CC=C2OCOC2=C1	10µM	**Increase**	Induction, Inhibition	28465217, 32039209
Phenethyl isothiocyanate	S=C=NCCC1=CC=CC=C1	2µM	decrease. Toxic to cells	Induction	19336571
ABT-737	CN(C)CC[C@H](CSC1=CC=CC=C1)NC1=CC=C(C=C1[N+]([O-])=O)S(=O)(=O)NC(=O)C1=CC=C(C=C1)N1CCN(CC2=C(C=CC=C2)C2=CC=C(Cl)C=C2)CC1	10µM	**Increase**	Induction	17446862
Lonafarnib	NC(=O)N1CCC(CC(=O)N2CCC(CC2)[C@H]2C3=C(CCC4=C2C(Br)=CC(Cl)=C4)C=C(Br)C=N3)CC1	10µM	Decrease	Induction	18769123
LY294002	O=C1C=C(OC2=C(C=CC=C12)C1=CC=CC=C1)N1CCOCC1	10µM	**Increase**	Induction, Inhibition	18330473, 9030745
Trazodone	ClC1=CC(=CC=C1)N1CCN(CCCN2N=C3C=CC=CN3C2=O)CC1	10µM	-	Induction	35781619
DL-Sulforaphane	CS(=O)CCCCN=C=S	10µM	-	Induction, Inhibition	24383989, 32476238
Dacinostat; LAQ824	OCCN(CCC1=CNC2=CC=CC=C12)CC1=CC=C(C=CC(=O)NO)C=C1	10µM	-	Induction	19383971
Selegiline	C[C@H](CC1=CC=CC=C1)N(C)CC#C	10µM	-	Inhibition	26342896
Carbamazepine	NC(=O)N1C2=C(C=CC=C2)C=CC2=C1C=CC=C2	10µM	**Increase**	Induction	23305067, 34432315
Concanavalin A		2µg/µL	-	Induction	17256764
Linoleic acid	CH3(CH2)4CH=CHCH2CH=CH(CH2)7CO2H	10µM	Decrease	Induction	32732901
